# A Bird-Watcher's Camp—I

**Published:** 1910-09-10

**Authors:** 


### The Practitioner's Relaxations.

### A BIRD-WATCHER'S CAMP—I.

The complete field-naturalist requires such an abundance of leisure as is vouchsafed to few of us; but a normally busy man can generally manage, with a little determination, to keep up a bowing acquaintance with Nature. In these days of week-ending, even a Londoner need not altogether despair —and then there are the parks. It must be admitted that town life deadens the subtle senses by which the "country-bumpkin" detects the flicker of the eye of a brooding partridge or the single unfamiliar note in the bird chorus at dawn, and feels rather than hears the faint rustle of a slinking bird in the hedgerow. But these senses are, I think, never killed outright except by persistent neglect; and fortunately the sense of hearing, invaluable to the bird-watcher, dies hardest and repays even very intermittent cultivation.

The most favoured of men are those whose daily work leads them abroad at all seasons of the year among the fields and hedgerows, woods and streams, moorlands and hills of the country. There are others less favoured, yet greatly blessed by comparison with the city working-man, whose work lies in the smaller county towns. Such was I before the prospect of a regular salary dragged me reluctant to London. My work lay in a little country town in the Midlands, and it kept me within doors during the normal working hours of the day. It followed that during the short days of the year I had comparatively scanty opportunities of indulging my fancy for watching birds, though I think I made the most of those I had. As regards the long days, when the birds are most active, when the summer migrants are here and the nursery business is in full swing, I am ashamed to remember how long it was before I thought of the obvious plan for making the most of them. It was not in fact till the last summer before I came to London that this plan occurred to me, and I can only take credit for having then put it into execution without delay.

### The Routine of Camp.

I was generally free at about half-past five in the afternoons, but I grudged the time wasted in reaching and returning from my favourite haunts, and revolted against the necessity of keeping comparatively regular hours for meals. The obvious remedy, which it took me so long to devise, was a camp among the birds. Here I could spend the long evenings and the early mornings, the Saturday half holiday and the Sunday day of rest untrammelled by any considerations of spoiled meals and the inconvenience caused to others by unpunctual habits. Having conceived this idea, I proceeded to act on it at once. A friend, to whom I am eternally grateful, gave me permission to camp by one of his game coverts. I purchased a small tent, a tarpaulin to sleep upon, and a few necessary utensils at a total cost of something less than £5, and I pitched my tent on June 11. I do not propose to dwell upon my camping arrangements. I must say, however, that matters were made very easy for me on this occasion by a friendly gamekeeper, who kept an eye generally on my belongings, and his wife, who relieved me as a rule from the necessity of washing up my crockery, etc. To the keeper, moreover, I was indebted for much interesting information imparted while he smoked an evening pipe at the door of my tent, or I accompanied him on his rounds. Two things always surprise me when I recall this time. The first I have already referred to—I cannot imagine why I did not think sooner of so obvious a plan for making the most of my time. The second is that I subsisted so cheerfully and without hurt on so small an allowance of sleep; for I woke regularly with the birds—*i.e.* at about 3 A.M.—and I seldom retired till long after the long days had come to an end. I am sometimes inclined to think that sleep is a mere habit—and a bad one.

### Dawning with the Birds.

I have heard people complain of being waked by the birds at dawn; for myself I ask nothing better. When I say that, on the first day after I pitched my tent, I found, in one small clump of bushes close by it, ten nests of various species of birds, and that later I found as many again in the same bushes, it will be realised that my fancy could hardly fail to be gratified.

The first bird I heard at 3 A.M. on my first morning in camp was a nightingale in full song close by. He was quickly followed by a robin and a thrush, and then, with this prelude, the great matutinal chorus—the prime—with which the birds salute the approaching day, broke out in full diapason—a medley of different songs in different keys, yet altogether free of discord. I was content for some time to lie where I was and listen, picking out the individual songs. Three cuckoos were calling without a symptom of a stammer, willow-wrens abounded, a single wren sang with vociferous energy, a blackbird chanted ballad music melodiously inconsequent, and a chiff-chaff reiterated his two notes with cheerful self-satisfaction. But every tree and bush was full of song, and my notes carry me no further in the enumeration of the individual songsters. This chorus is of daily occurrence, and nothing quite like it is to be heard for the rest of the day. It begins and ends suddenly. After about an hour comparative silence falls; the day's work has begun—the serious business of seeking food, building nests, and tending the young. And whether it is because they are so particularly busy I do not know, but there is no better time than the very early morning in all the day for watching birds. They are never so approachable as then, and, paddling about in the dew-laden grass, with unstockinged feet thrust into an old pair of tennis shoes, I have found myself on such friendly terms with them as I knew that, later in the day, they would not tolerate.

The Results of a Month's Observation.

I kept my camp in being for a calendar month exactly, striking my tent with profound regret on July 11, because my duties called me to another part of the country. During this period I spent only occasional nights under the shelter of a roof when late work detained me. Never did time pass so happily; I do not remember a single dull moment in it. It must not be supposed, however, that I made any great and startling discoveries. I learned much; but I cannot claim to have added anything of moment to the stock of zoological or ornithological knowledge in the world. I was merely improving my acquaintance with some of our common birds and enjoying myself thoroughly in the process. I propose to recount, as they occur to me, some of the observations which interested me most, for the sake of the pleasure it gives me to recall them and the pleasure that others may find in comparing them with their own experience.

*(To be continued.)*

